# Insights into distinct regulatory modes of nucleosome positioning

**DOI:** 10.1186/1471-2164-10-602

**Published:** 2009-12-14

**Authors:** Zhiming Dai, Xianhua Dai, Qian Xiang, Jihua Feng, Yangyang Deng, Jiang Wang

**Affiliations:** 1 Electronic Department, Sun Yat-Sen University, Guangzhou, China

## Abstract

**Background:**

The nucleosome is the fundamental unit of eukaryotic genomes. Experimental evidence suggests that the genomic DNA sequence and a variety of protein factors contribute to nucleosome positioning *in vivo*. However, how nucleosome positioning is determined locally is still largely unknown.

**Results:**

We found that transcription factor binding sites (TFBSs) with particular nucleosomal contexts show a preference to reside on specific chromosomes. We identified four typical gene classes associated with distinct regulatory modes of nucleosome positioning, and further showed that they are distinguished by transcriptional regulation patterns. The first mode involves the cooperativity between chromatin remodeling and stable transcription factor (TF)-DNA binding that is linked to high intrinsic DNA binding affinities, evicting nucleosomes from favorable DNA sequences. The second is the DNA-encoded low nucleosome occupancy that is associated with high gene activity. The third is through chromatin remodeling and histone acetylation, sliding nucleosomes along DNA. This mode is linked to more cryptic sites for TF binding. The last consists of the nucleosome-enriched organization driven by other factors that overrides nucleosome sequence preferences. In addition, we showed that high polymerase II (Pol II) occupancy is associated with high nucleosome occupancy around the transcription start site (TSS).

**Conclusions:**

We identified four different regulatory modes of nucleosome positioning and gave insights into mechanisms that specify promoter nucleosome location. We suggest two distinct modes of recruitment of Pol II, which are selectively employed by different genes.

## Background

The nucleosome is the basic repeating unit of eukaryotic chromatin, consisting of a histone octamer around which 147 DNA base pairs are wrapped. DNA wrapped in nucleosomes is less accessible than linker DNA, nucleosome positioning thus plays a profound role in transcription by controlling access of genomic DNA to most DNA binding proteins. The generation of high-resolution nucleosome maps in several organisms helps the understanding of the genome-wide organization of nucleosomes and its relationship with transcriptional regulation [1-8]. In general, the level of nucleosome occupancy in promoter is inversely proportional to the corresponding gene transcription rate. Recent studies have revealed that the presence of nucleosomes close to the transcription start site (TSS) is associated with variation in gene expression [9,10].

The coordination of nucleosome positions is a complex process involving combined interactions among multiple factors. Experimental evidence indicates that the intrinsic DNA sequence is one dominant determinant of nucleosome positioning [11]. Several studies have used DNA sequence features to predict genome-wide nucleosome positions with modest success [12-16], suggesting that nucleosome positioning is partly encoded in the genomic DNA sequence itself. Moreover, changes in the DNA-encoded nucleosome organization are linked to distinct transcriptional programs [17] and evolutionary changes in gene expression [18]. On the other hand, factors other than the surrounding DNA sequence also contribute to nucleosome positioning. For example, the chromatin remodeling complex Isw2 can override the underlying DNA sequence to reposition nucleosomes, suppressing transcription initiation from cryptic sites [3].

The transcriptional program is controlled by binding of transcription factors (TFs) to the specific DNA sequences in promoter regions upstream of the genes they regulate. TF binding shows a preference for nucleosome-depleted regions [19,20]. However, a considerable fraction of TFBSs reside in nucleosomes rather than in linker DNA [5,7]. Previous studies have revealed that the location, orientation and spacing of transcription factor binding sites (i.e. the contexts of TFBSs) can affect gene expression [21,22], this leaves open the question of whether different nucleosomal contexts of TFBSs have distinct effects on gene regulation. We refer to nucleosome occupancy and its determinants (the underlying DNA sequence and other factors) as nucleosomal context.

Although several factors are responsible for governing nucleosome positioning along the genome, it is less clear how these determinants work in concert to regulate nucleosome positioning. The origins and consequences of different regulatory modes of nucleosome positioning also remain to be elucidated. Recently, the generated genome-wide occupancy of nucleosomes assembled on purified yeast genomic DNA [23] makes it possible to address these issues. Analyzing these data along with other available data, we found that nucleosomal contexts of TFBSs differ across chromosomes. We further identified four distinct regulatory modes of nucleosome positioning: DNA-encoded open nucleosome (nucleosome-depleted) organization, nucleosome eviction, nucleosome sliding, and non-DNA-driven closed nucleosome (nucleosome-enriched) organization. Our results reveal that the modes of nucleosomal regulation are linked to the properties of transcription factor binding motifs. Furthermore, the four modes of nucleosomal regulation are associated with distinct gene regulation patterns. Our results indicate that the nucleosomal context of TFBSs affects the regulatory function of TFs on their target genes. We also found distinct patterns of RNA polymerase II (Pol II) occupancy associated with nucleosome occupancy around the TSS.

## Results

### Transcription factor binding sites with different nucleosomal contexts

For each TFBS experimentally identified in YPD medium [24], we used two types of nucleosome occupancy data [23] to determine its nucleosomal context. One is *in vivo *nucleosome occupancy measured in YPD medium, the other is *in vitro *nucleosome occupancy governed only by the intrinsic sequence preferences of nucleosomes. The *in vivo *nucleosome occupancy represents the combined effects of multiple nucleosome positioning determinants (e.g. DNA sequences, chromatin remodeling activities), while the *in vitro *nucleosome occupancy indicates the nucleosome sequence preferences alone. For either nucleosome map, we classified nucleosome-enriched regions from nucleosome-depleted regions as described in the original literature [23]. We clustered TFBSs into four groups according to their locations (nucleosome-enriched regions or nucleosome-depleted regions): nucleosome-enriched *in vitro *but nucleosome-depleted *in vivo *(vitro+/vivo-), nucleosome-depleted *in vitro *and *in vivo *(vitro-/vivo-), nucleosome-enriched *in vitro *and *in vivo *(vitro+/vivo+), nucleosome-depleted *in vitro *but nucleosome-enriched *in vivo *(vitro-/vivo+). Moreover, we examined the four clusters on an additional dataset of *in vivo *nucleosome occupancy in YPD medium [1]. Indeed, nucleosome occupancy around vitro+/vivo- and vitro-/vivo- TFBSs is significantly lower than that around vitro+/vivo+ and vitro-/vivo+ TFBSs (*P *≈ 0, Mann-Whitney U-test; see Additional file 1). DNA is more likely to encode nucleosome occupancy around vitro-/vivo- and vitro+/vivo+ TFBSs *in vivo*, as they have similar nucleosome occupancy both *in vivo *and *in vitro*, respectively (see Additional file 2). On the other hand, other factors rather than DNA tend to determine nucleosome occupancy around vitro+/vivo- and vitro-/vivo+ TFBSs *in vivo*, for they show significant changes in nucleosome occupancy between *in vivo *and *in vitro*, respectively (see Additional file 2).

### TFBSs with particular nucleosomal contexts tend to reside on specific chromosomes

**Figure 1:**
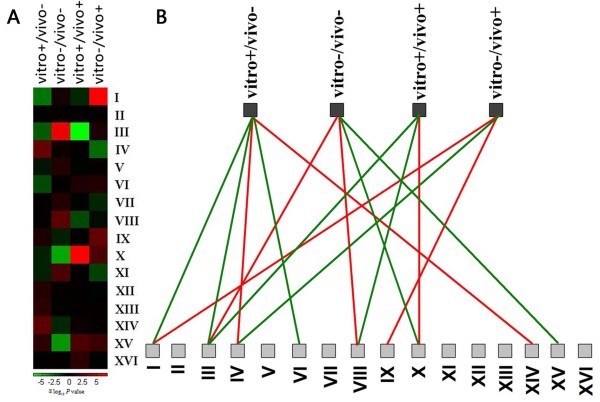
**Chromosomal preference and dispreference for each TFBS class**. (A) Rows represent the 16 chromosomes labelled I to XVI, and each column represents the *μ *log_10 _*P *value (negative sign indicates that the number of TFBSs is higher than expected (i.e. preference), while positive sign indicates that the number of TFBSs is lower than expected (i.e. dispreference)) significance profile of a specific TFBS class. For each of the four TFBS classes, we first counted the numbers of TFBSs that are on each of the 16 chromosomes and on the other 15 chromosomes, respectively. We next counted the total numbers of all TFBSs that are on each of the 16 chromosomes and on the other 15 chromosomes, respectively. By using chi-test to evaluate the overlap in membership of specific class of TFBSs (observed occurrence) with the collection of all TFBS classes (expected occurrence) that reside on the specific chromosome and on the other chromosomes, we evaluated whether the particular TFBS class shows a strong preference to reside or is uniformly distributed on specific chromosome. *P *values were calculated using the CHITEST formula in Excel. (B) The favoured and disfavoured chromosomes for each of the 4 TFBS classes. The top column denotes the 4 TFBS classes, and the bottom column denotes the 16 chromosomes. Red and green lines connecting the two columns mean that the TFBS classes tend to preferentially reside or avoid residing on a particular chromosome (*P *< 0.01), respectively.

We asked whether TFBSs with particular nucleosomal contexts are uniformly distributed across different chromosomes or encoded on specific chromosomes. We first created a chromosome preference profile for each of the four TFBS classes, which is a vector that contains the number of TFBSs on each of the 16 chromosomes. By comparing this vector with what is observed by all TFBS classes, we evaluated whether the particular TFBS class tends to reside on specific chromosomes. We found that all the four TFBS classes show a strong preference for specific chromosomes (*P *< 10^-6 ^for vitro+/vivo- TFBSs, *P *< 10^-10 ^for vitro-/vivo- TFBSs, *P *< 10^-9 ^for vitro+/vivo+ TFBSs, *P *< 10^-8 ^for vitro-/vivo+ TFBSs, Chi-test). We next examined which chromosomes are significantly favoured or disfavoured by each of the four TFBS classes, using Chi-test to evaluate the overlap in membership of specific class of TFBSs with the collection of TFBSs that reside on the specific chromosome. Our analysis revealed that all TFBS classes have their own favoured and disfavoured chromosomes (Figure 1). For example, vitro-/vivo- TFBSs show a preference on chromosome III (*P *< 10^-6^) and VIII (P < 10^-2^), but show a dispreference on chromosome X (*P *< 10^-4^) and XV (*P *< 10^-4^). In contrast, vitro+/vivo+ TFBSs tend to reside on X (*P *< 10^-6^), but less reside on chromosome III (*P *< 10^-6^) and VIII (*P *< 10^-2^). These results demonstrate that nucleosomal contexts of TFBSs differ rather than be uniform across the chromosomes. To test whether these differences are due to telomeric heterochromatin, we next examined whether TFBSs with particular nucleosomal contexts tend to preferentially locate or avoid on specific regions on the linear chromosomes, such as regions closer to the centromere or the telomere, or the regions in-between. We found that the four TFBS classes show no significant preference or avoidance to reside on specific regions on the chromosomes (see Additional file 3). In addition, vitro+/vivo- and vitro-/vivo- TFBSs show a strong preference for regions immediately upstream of genes, whereas vitro+/vivo+ and vitro-/vivo+ TFBSs are distributed more uniformly throughout promoters (see Additional file 4). This results are consistent with the observations that a well-known substantial nucleosome-free region (NFR) exists directly upstream of the TSS [1], and TFBSs tend to reside in regions depleted of nucleosomes [5,19].

### High intrinsic TF-DNA binding affinity overcomes nucleosomal barrier

**Figure 2:**
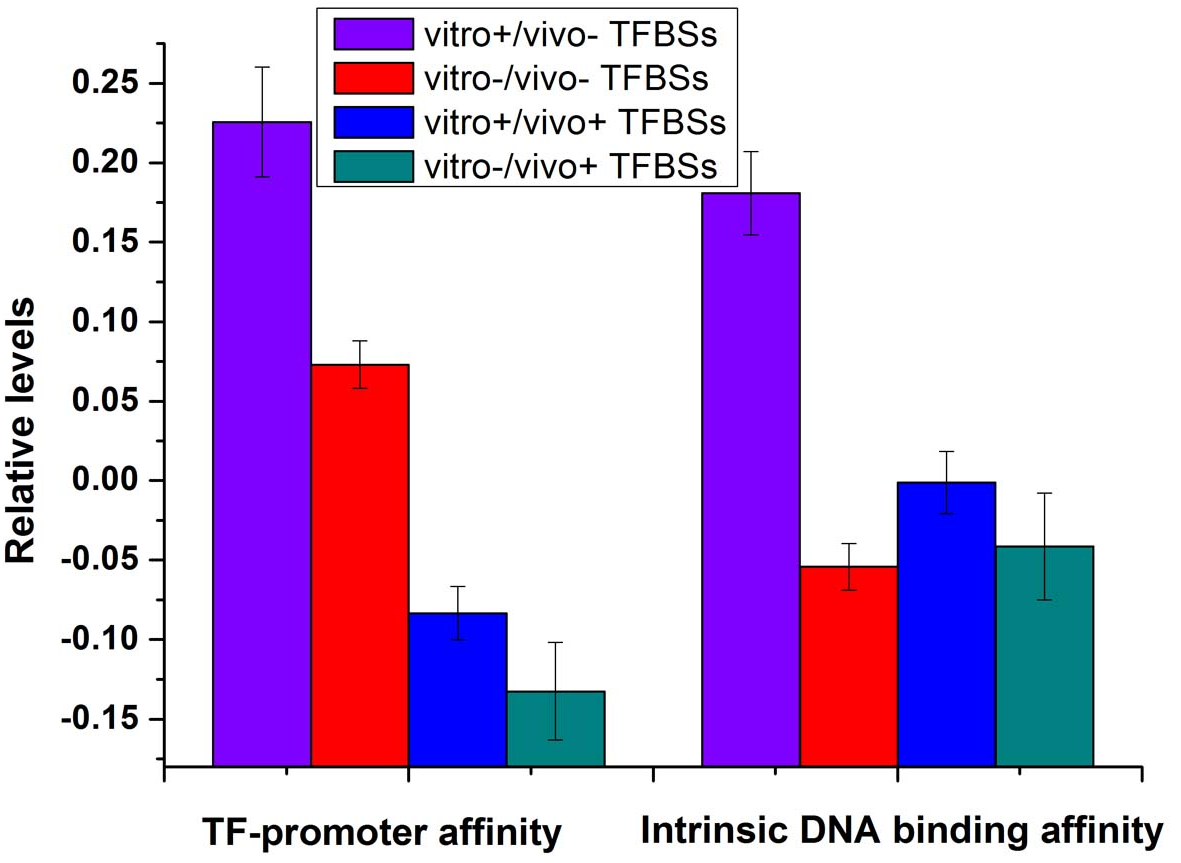
**TF binding affinities for the four classes**. Average values that correspond to experimentally measured TF-promoter binding affinity [24], and intrinsic DNA binding affinity calculated from PWM are shown for the four TFBS classes. Values in each property were normalized, such that their means are zero and standard deviations are one. Error bars were calculated by bootstrapping.

We asked whether different nucleosomal contexts of TFBSs have distinct effects on TF binding. Previous studies have shown that TFs bind specific sequences not only on linker DNA but also on nucleosomes [5,7,19,20,25], but whether there is difference in TF binding affinities between these two situations remains to be answered. Using the genome-wide experimentally measured TF-promoter binding data [24], we found that vitro+/vivo- and vitro-/vivo- TFBSs have significantly higher experimental TF binding affinities than the other two types of TFBSs (*P *< 10^-17^, Mann-Whitney U-test; Figure 2). This result demonstrates that high TF binding affinities are generally associated with TFBSs that reside on nucleosome-depleted regions.

Although both vitro+/vivo- and vitro-/vivo- TFBSs are depleted of nucleosomes *in vivo*, vitro+/vivo- TFBSs have significantly higher TF binding affinities than vitro-/vivo- TFBSs (*P *< 10^-7^, Mann-Whitney U-test; Figure 2). As nucleosome occupancy governed only by DNA (*in vitro*) is high at vitro+/vivo- TFBSs, we speculated that the high-affinity TF binding contributes to the *in vivo *low nucleosome occupancy around vitro+/vivo- TFBSs. If this is the case, vitro+/vivo- TFBSs might have high intrinsic DNA binding affinities that ensure high-affinity TF binding *in vivo *to overcome nucleosomal barrier. To test this possibility, we scored each TFBS for a match to the corresponding position weight matrix (PWM) [24]. The resulting score is known to provide reasonable approximation for the intrinsic affinity of specific DNA sequence for the TF [26]. Vitro+/vivo- TFBSs indeed have significantly higher intrinsic DNA binding affinities than the other TFBSs (*P *< 10-^14^, Mann-Whitney U-test; Figure 2). We also found that vitro+/vivo- TFBSs are slightly enriched in essential genes [27] (*P *= 0.007, Chi-test). A natural question arises concerning whether the high intrinsic DNA binding affinity is the hallmark of essential genes. But we found that the intrinsic DNA binding affinities of essential genes are comparable to those of the other genes (*P *= 0.6, Mann-Whitney U-test).

### Distinct regulatory modes of nucleosome positioning

**Figure 3:**
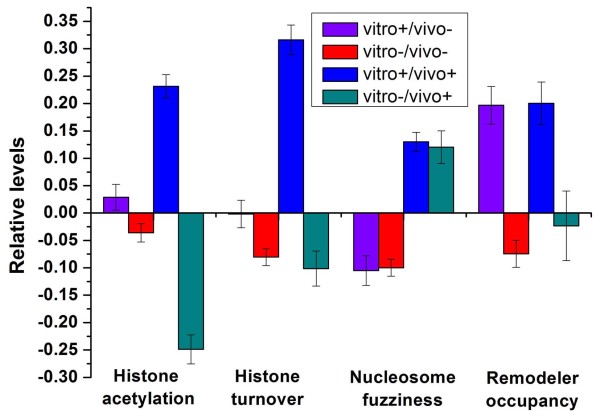
**Nucleosomal features that distinguish the four classes**. Average values that correspond to mean histone acetylation levels [29] (H3K9, H3K14, and H4 acetylation), the turnover rate of H3 histone [30], nucleosome fuzziness [4], and the mean levels of the occupancy for the seven remodelers [31] are shown for the four classes. For properties except remodeler occupancy, we assigned each TFBS with the corresponding value of its nearest probe. For remodeler occupancy, we assigned each promoter with the corresponding value. Values in each property were normalized, such that their means are zero and standard deviations are one. Error bars were calculated by bootstrapping.

One way in which cells modulate nucleosomes is histone modification. Different from other known modifications, acetylation can neutralize the positive charge of the lysine. As a result, acetylated histone tails are thought to associate more loosely with nucleosomal DNA than unmodified and methylated histone tails [28]. A previous study has profiled three types of histone acetylation across the yeast genome with high resolution [29]. We found that regions around vitro+/vivo+ TFBSs are characterized by hyperacetylation (*P *< 10^-37^, Mann-Whitney U-test; Figure 3). A recent study has proposed that replacement of nucleosomes with appropriately modified histones in nucleus helps to maintain proper patterns of histone modification [30]. Indeed, nucleosomes around vitro+/vivo+ TFBSs have significantly higher rates of histone H3 turnover [30] (number of H3 replacement events per unit of time) than those around the other TFBSs (*P *< 10^-53^, Mann-Whitney U-test; Figure 3). Nucleosome sliding and eviction are two important ways of regulating access to DNA. Sliding the histone octamer makes the nucleosome spread out over a broad region along DNA. Consequently, it should cause nucleosomes delocalization along DNA. We used nucleosome fuzziness to represent nucleosome delocalization as in a previous study [4]. Nucleosomes around vitro+/vivo+ and vitro-/vivo+ TFBSs are more delocalized than those around vitro+/vivo- and vitro-/vivo- TFBSs (*P *< 10^-25^, Mann-Whitney U-test; Figure 3). Along with the above observation that vitro+/vivo+ and vitro-/vivo+ TFBSs are enriched with nucleosomes *in vivo*, that is, nucleosomes are not widely evicted around these TFBSs, we suggest that nucleosome sliding is a prevalent way for regulating access to them.

Chromatin remodeling is another way of nucleosome modulation. A recent study has measured occupancy at every yeast promoter for seven chromatin remodelers (Isw1a, Isw1b, Isw2, Swi/Snf, Rsc, Ino80, and Swr-c) [31], yielding opportunity for new insight into the mechanisms of chromatin remodeling activities. As the remodeler occupancy was measured with individual promoter resolution, we identified four promoter clusters in terms of the types of their TFBSs. To avoid confusion, we restricted the analysis to promoters containing only one type of TFBSs (266 vitro+/vivo- promoters, 887 vitro-/vivo- promoters, 252 vitro+/vivo+ promoters, 102 vitro-/vivo+ promoters, see Additional file 5), that is, we excluded promoters containing two or more types of TFBSs. Vitro+/vivo- promoters have closed nucleosome organization *in vitro *but relatively open nucleosome organization *in vivo*, whereas vitro-/vivo+ promoters show relatively open nucleosome organization *in vitro *but closed nucleosome organization *in vivo *(see Additional file 6). These results reveal that change in nucleosome occupancy between *in vivo *and *in vitro *not only occur around TFBSs, but also in broader regions at vitro+/vivo- and vitro-/vivo+ promoters. Vitro-/vivo- promoters have low nucleosome occupancy both *in vivo *and *in vitro *(see Additional file 6), their downstream genes have higher gene activities than genome-wide level in terms of transcription rates [32] (11.8 versus 6.7; *P *< 10^-8^, Mann-Whitney U-test).

We next analyzed the remodeler occupancy for the four types of promoters (Figure 3). As vitro+/vivo- promoters have significantly lower nucleosome occupancy *in vivo *compared with that *in vitro*, they are expected to be highly regulated by chromatin remodelers. Indeed, vitro+/vivo- promoters have significantly higher remodeler occupancy than genome-wide level (*P *< 10^-5^, Mann-Whitney U-test). Considering that vitro+/vivo- promoters have significantly lower nucleosome occupancy *in vivo *compared with *in vitro*, high remodeler occupancy, and low nucleosome delocalization, these observations indicate that nucleosome eviction rather than nucleosome sliding is required to permit access to vitro+/vivo- TFBSs. Although vitro+/vivo+ promoters have similar closed nucleosome organization both *in vivo *and *in vitro*, they are shown to be highly regulated by chromatin remodelers (*P *< 10^-5^, Mann-Whitney U-test). Together with the above observation that vitro+/vivo+ promoters tend to be regulated by histone acetylation, we suggest that chromatin remodeling work in concert with histone acetylation to modulate nucleosome positioning at vitro+/vivo+ promoters. As expected, vitro-/vivo- promoters, largely depleted of nucleosomes *in vivo *and *in vitro*, are less dependent on chromatin remodelers (*P *< 10^-5^, Mann-Whitney U-test). This result further confirms that nucleosome occupancy at vitro-/vivo- promoters is mainly determined by the DNA. Vitro-/vivo+ promoters show increased nucleosome occupancy *in vivo *compared with *in vitro*, but they are not significantly involved in chromatin remodeling (*P *= 0.6, Mann-Whitney U-test). More specifically, vitro-/vivo+ promoters are not characterized by high occupancy for any of the seven chromatin remodelers (*P *> 0.05, Mann-Whitney U-test), indicating that other remodelers or factors govern nucleosome occupancy at vitro-/vivo+ promoters.

Taken together, we identified distinct regulatory modes of nucleosome positioning. Vitro+/vivo- promoters employ nucleosome eviction for chromatin remodeling, whereas vitro+/vivo+ promoters use nucleosome sliding and histone acetylation to regulate nucleosome activities. The open nucleosome organization at vitro-/vivo- promoters is mainly encoded in DNA.

### DNA-encoded closed nucleosome organization protects unbound motifs

As shown before, vitro+/vivo+ promoters have similar closed nucleosome organizations both *in vivo *and *in vitro *(see Additional file 6). In addition, they employ nucleosome sliding rather than nucleosome eviction to retain the closed nucleosome organization and regulate access to DNA. We sought to understand why they use this mode of nucleosomal regulation. It is well-known that transcription factor binding motifs are short and degenerate, unbound motifs are thus widely distributed throughout the genome. We speculated that the DNA-encoded closed nucleosome organization protects unbound motifs at vitro+/vivo+ promoters. Using the dataset of unbound motifs identified in a previous study [24], we found that vitro+/vivo+ promoters are highly enriched with unbound motifs than genome-wide level (*P *< 10^-6^, Mann-Whitney U-test). Since intergenic distances in the yeast genome are not very large, the inappropriate access to unbound motifs located upstream of two divergently transcribed genes may simultaneously affect gene expression of both genes. We identified genes as divergent genes if their 5'end intergenic regions are divergent, and the other genes as tandem genes. Our result shows that vitro+/vivo+ promoters tend to be divergent genes compared with genome-wide level (*P *< 10^-6^, Chi-test).

### Distinct transcriptional programs by nucleosomal regulation

**Figure 4:**
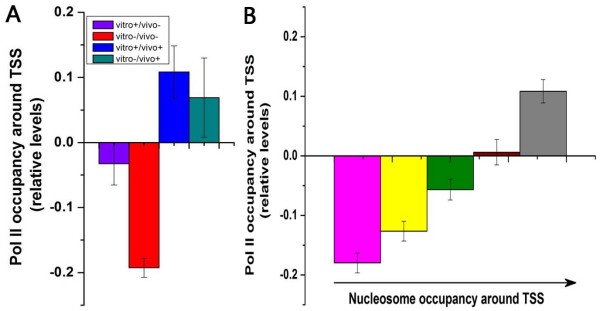
**Relationship between Pol II occupancy and nucleosome occupancy around TSS**. (A) Pol II occupancy [33] around TSS (from -100 to +100) are shown for the four gene classes. Values in each property were normalized, such that their means are zero and standard deviations are one. (B) All genes were divided into five groups according to their average nucleosome occupancy [23] around TSS (from -100 to +100), and the average Pol II occupancy around TSS was shown for each group. Error bars were calculated by bootstrapping.

The assembly of Pol II is an important step in transcription initiation. We found that vitro+/vivo+ and vitro-/vivo+ promoters are enriched with Pol II [33] around TSS (*P *< 10^-3^, Mann-Whitney U-test), whereas vitro-/vivo- promoters are depleted of Pol II around TSS (*P *< 10^-26^, Mann-Whitney U-test; Figure 4A). Given that the four types of promoters show significant difference in nucleosome occupancy immediately upstream of genes (see Additional file 6), we speculated that Pol II occupancy is associated with nucleosome occupancy around TSS. As expected, high nucleosome occupancy corresponds to high Pol II occupancy around TSS (Figure 4B). We reasoned that high Pol II occupancy around TSS indicates pre-engaged Pol II, which could facilitate the recruitment of Pol II at highly nucleosome-occupied promoters upon transcriptional activation (see Discussion).

We next examined whether nucleosomal context of TFBSs is linked to TF regulation. In fact, mere TF binding is not sufficient to guarantee its regulation. Genes coregulated by a given TF are expected to be coexpressed. We asked whether nucleosomal context of TFBSs influences gene coexpression. We used a combined gene expression data set in 255 conditions covering environmental stresses [34] and cell cycle [35]. For each TF, we calculated the pair-wise Pearson correlation coefficients among expression profiles of its target genes (i.e. the TF cohort). We restricted the analysis to TFs with more than 20 target genes. Moreover, the pair-wise Pearson correlation coefficients for expression profiles of each TF cohort were computed for each type of genes (e.g. vitro+/vivo- genes). We showed that the average coefficient for vitro-/vivo- genes alone is significantly higher the other three types (*P *< 10^-23^, Mann-Whitney U-test), suggesting that the DNA-encoded low nucleosome occupancy around TFBSs contributes to the regulatory function of TFs.

## Discussion

It has become evident that nucleosome sequence preferences and other factors regulate nucleosomal organization *in vivo*. However, how nucleosome positioning is regulated locally remains unclear. Using multiple sources of data generated in YPD medium, we identified four typical promoter classes characterized by distinct regulatory modes of nucleosome positioning. Insights into the four modes of nucleosomal regulation reveal that DNA encodes closed nucleosome organization to occlude cryptic sites between some divergent gene pairs, and high intrinsic TF-DNA binding affinity is associated with nucleosome displacement that overrides the underlying nucleosome sequence preferences. We also found that the four modes differ in Pol II occupancy around TSS and coexpression of TF target genes.

A key finding of this study is that Pol II occupancy is correlated with nucleosome occupancy around TSS. A recent study has found that nucleosome occupancy around TSS is correlated with transcriptional plasticity [9], which quantifies the dynamic range of expression level in various conditions (see Materials and methods section for details). However, we found that there is no significant link between transcriptional plasticity and Pol II occupancy around TSS (see Additional file 7). The correspondence between Pol II occupancy and nucleosome occupancy around TSS suggests two distinct modes of recruitment of Pol II. As DNA may be transiently accessible at promoters with high nucleosome occupancy around TSS, Pol II should be pre-engaged around TSS for transcription activation. On the other hand, pre-engaged Pol II seems unnecessary for promoters with low nucleosome occupancy around TSS, as their open chromatin structure facilitates regulatory proteins binding which can subsequently recruit Pol II for transcription.

Although DNA at vitro+/vivo- promoters encodes closed nucleosome organization *in vitro*, the nucleosome organization becomes relatively open *in vivo*. We found that this drastic change in nucleosome occupancy is due to two reasons. First, the high remodeler occupancy indicates that much chromatin remodeling is involved in nucleosome positioning at vitro+/vivo- promoters. As they do not show high nucleosome delocalization which is the hallmark of nucleosome sliding, we suggest that nucleosome eviction is the main mode of chromatin remodeling at vitro+/vivo- promoters. Second, the high intrinsic DNA binding affinities at vitro+/vivo- promoters enhances the capacity of TFs to compete with nucleosomes for occupancy along DNA, probably resulting in the low nucleosome occupancy *in vivo*. It will be very interesting to understand how the high nucleosome sequence preferences, the high intrinsic TF-DNA binding affinities, and chromatin remodelers together determine nucleosome dynamics at vitro+/vivo- promoters.

Vitro+/vivo+ promoters are enriched with unbound motifs. Their closed nucleosome organization encoded in DNA seems to protect these cryptic sites. They employ nucleosome sliding and histone acetylation to affect the interaction of histones with DNA. As our analysis is conducted in YPD medium, it is very interesting to investigate whether this regulatory mode of nucleosome positioning is conserved in other cellular conditions. They have lower TF-promoter affinities than vitro+/vivo- and vitro-/vivo- promoters (*P *< 10^-9^, Mann-Whitney U-test; Figure 2). This relatively unstable TF binding which may be due to their high nucleosome occupancy and delocalization, might limit the regulatory function of TFs.

Vitro-/vivo- promoters, which are less dependent on chromatin remodeling, have open nucleosome organization encoded in DNA. This organization might guarantee the recruitment of TFs to activate transcription. As a result, the corresponding genes of vitro-/vivo- promoters have high gene activities. Other factors override the underlying DNA sequence to form the closed nucleosome organization at vitro-/vivo+ promoters. On the other hand, they show high nucleosome delocalization. A possible explanation is that the competition between TFs and nucleosomes cause nucleosome delocalization.

## Conclusions

Eukaryotic genomes are packaged into chromatin, the major structural element of which is nucleosome. Nucleosome positioning plays an important role in diverse cellular processes that rely on access to genomic DNA. The genomic DNA sequence and regulatory proteins are the dominant determinants of nucleosome positioning, but how they determine nucleosome positioning locally and the subsequent influences on transcriptional regulation remain elusive. We found that transcription factor binding sites (TFBSs) with particular nucleosomal contexts show a preference to reside on specific chromosomes. Furthermore, we identified four promoter classes characterized by distinct regulatory modes of nucleosome positioning: DNA-encoded open nucleosome organization, nucleosome eviction, the cooperativity between nucleosome sliding and histone acetylation, and non-DNA-driven closed nucleosome organization. These four modes are linked to the properties of transcription factor binding motifs, and are associated with distinct transcriptional regulation patterns. We found the relationship between polymerase II (Pol II) occupancy and nucleosome occupancy around the transcription start site (TSS), which suggests two distinct modes of recruitment of Pol II.

## Methods

### Identification of four TFBS classes according to their nucleosomal context

Transcription factor binding data was taken from Harbison *et al. *[24], which includes the binding affinities of various TFs to all promoters in YPD medium. They also indentified exact binding sites at promoters for each TF. They also calculated PWM from binding sites for each of TF. A *P *value cutoff of 0.001 was used to define the set of genes bound by a particular TF. By applying this strict binding threshold, we ensured a low level of false positives. The data set includes 9,678 binding sites for 101 TFs. We also scored each TFBS for a match to the corresponding PWM. The resulting score indicates the intrinsic TF-DNA binding affinity.

Genome-wide nucleosome occupancy data *in vivo *and *in vitro *were measured with 1-bp resolution by Kaplan *et al. *[23]. They used statistical methods to define nucleosome-enriched regions and nucleosome-depleted regions. Using their definition, we clustered TFBSs into four groups according to their locations (nucleosome-enriched regions or nucleosome-depleted regions): nucleosome-enriched *in vitro *but nucleosome-depleted *in vivo *(vitro+/vivo-), nucleosome-depleted *in vitro *and *in vivo *(vitro-/vivo-), nucleosome-enriched *in vitro *and *in vivo *(vitro+/vivo+), nucleosome-depleted *in vitro *but nucleosome-enriched *in vivo *(vitro-/vivo+). We also validated our classification using another genome-wide data set of nucleosome occupancy measured with 4-bp resolution in YPD medium [1]. We calculated for each TFBS the average nucleosome occupancy across the region it covers, and found that nucleosome occupancy around vitro+/vivo- and vitro-/vivo- TFBSs is significantly lower than that around vitro+/vivo+ and vitro-/vivo+ TFBSs.

We mapped binding sites to the corresponding genes according to their located promoters (1,000 bp upstream of the gene in this study, the upstream region was truncated if it overlapped with neighboring genes), and then mapped these TFBSs with binding affinities of the associated TFs to the corresponding promoters. If the binding sites locate between divergent gene pairs, we mapped the binding sites to their nearest genes. We further clustered TFBS-located promoters into four groups according to the clusters which their TFBSs belong to: vitro+/vivo- promoters, vitro-/vivo- promoters, vitro+/vivo+ promoters, and vitro-/vivo+ promoters. To avoid confusion, we restricted the analysis to promoters containing only one type of TFBSs, that is, we excluded promoters containing two or more types of TFBSs.

### Gene expression data

The transcription rates were taken from Holstege et al. [32], at the condition similar to that where nucleosome occupancy was measured, which were normalized, such that their means are zero and standard deviations are one. Gene expression data used for coexpression analysis was measured in environmental stresses [34] and cell cycle [35], a total of 255 conditions. Using the highly significant (*P *≤ 0.001) binding data [24], we calculated for each TF the pair-wise Pearson correlation coefficients among expression profiles of its target genes (i.e. the TF cohort). In addition, we also repeated the calculation for vitro+/vivo- genes alone, vitro-/vivo- genes alone, vitro+/vivo+ genes alone, and vitro-/vivo+ genes alone, respectively. We compiled available gene expression data from the Stanford Microarray Database [36], a total of 1,260 published microarray experiments for 6,260 genes in various cellular conditions. For each gene, we calculated the averaged of the squared expression level from the 1,260 experiments as described in a previous study [9], and defined the normalized resulting value as transcriptional plasticity, which reflected the dynamic extent of its expression level in various conditions.

### Nucleosome-related data

Chromatin remodeler occupancy at TSS and UAS, including Isw1a, Isw1b, Isw2, Swi/Snf, Rsc, Ino80, and Swr-c, were taken from Venters *et al. *[31], which were normalized for each remodeler, such that their means are zero and standard deviations are one. Histone acetylation data (including H3K9ac, H3K14ac and H4ac) across the yeast genome were taken from Pokholok *et al. *[29]. We assigned each TFBS with the acetylation level of its nearest probe. Turnover rates of histone H3 were taken from Dion *et al. *[30]. We assigned each TFBS with the turnover rate of its nearest probe. The nucleosome fuzziness data was taken from Mavrich *et al. *[4]. We assigned each TFBS with the fuzziness of its nearest nucleosome.

### Other data

Gene coordinate data were downloaded from the Saccharomyces Genome Database [37]. The TSS data was taken from David *et al. *[38]. Pol II occupancy data were taken from Steinmetz *et al. *[33]. We calculated for each gene the average Pol II and nucleosome occupancy around TSS (from -100 to +100). Unbound motif data (files 'nobind_c3.gff') were taken from Harbison *et al. *[24]. Likewise, we mapped these unbound motifs to the corresponding genes according to their located promoters. A list of essential genes was taken from Winzeler et al. [27].

## Authors' contributions

ZD and XD analyzed the results and drafted the manuscript, and ZD also designed the study, implemented the algorithms, carried out the experiments. QX, JF, YD and JW participated in the analysis and discussion. All authors read and approved the final manuscript.

## Supplementary Material

Additional file 1 ***In vivo *nucleosome occupancy by Lee et al. for the four TFBS classes**. Average values that quantify the levels of *in vivo *nucleosome occupancy [1] are shown for the four TFBS classes. We calculated for each TFBS the average nucleosome occupancy over the region it covers. Error bars were calculated by bootstrapping. Click here for file

Additional file 2 ***In vitro *and *in vivo *nucleosome occupancy by Kaplan et al. for the four TFBS classes**. The average *in vitro *and *in vivo *nucleosome occupancy [23] over each TFBS was computed, respectively. The average resulting values are shown for the four TFBS classes (1,378 vitro+/vivo- TFBSs, 4,235 vitro-/vivo- TFBSs, 2,377 vitro+/vivo+ TFBSs, 980 vitro-/vivo+ TFBSs). Error bars were calculated by bootstrapping. Click here for file

Additional file 3 **Regional preference and dispreference for each TFBS class**. Rows represent the 3 regions labelled telomere (the ends of chromosomes, 5% of the chromosomes), centromere (taken from SGD [37]) and the middle region between centromere and telomere. Each column represents the μ log_10 _*P *value (negative sign indicates that the number of TFBSs is higher than expected (i.e. preference), while positive sign indicates that the number of TFBSs is lower than expected (i.e. dispreference)) significance profile of a specific TFBS class. For each of the four TFBS classes, we first counted the numbers of TFBSs that are on each of the 3 regions and on the other 2 regions, respectively. We next counted the total numbers of all TFBSs that are on each of the 3 regions and on the other 2 regions, respectively. By using chi-test to evaluate the overlap in membership of specific class of TFBSs (observed occurrence) with the collection of all TFBS classes (expected occurrence) that reside on the specific region and on the other regions, we evaluated whether the particular TFBS class shows a strong preference or avoidance to reside on specific region. *P *values were calculated using the CHITEST formula in Excel. Using the threshold (*P *< 0.01) to assess the statistical significance, we found that only vitro-/vivo+ TFBSs showed a slight preference to telomere (*P *= 0.008). The other three TFBS classes did not show a preference or avoidance to reside on specific regions on the chromosomes. Click here for file

Additional file 4 **Distribution of TFBSs at promoter regions**. Distribution of TFBSs relative to the ATG start codon of an ORF is shown for the four TFBS classes. Click here for file

Additional file 5 **Table S5**. ORF names for vitro+/vivo- promoters, vitro-/vivo- promoters, vitro+/vivo+ promoters, and vitro-/vivo+ promoters. Click here for file

Additional file 6 **Average nucleosome occupancy at promoter regions**. (A) Average nucleosome occupancy *in vivo *[23] is shown for the four promoter classes and all promoters. (B) Average nucleosome occupancy *in vitro *[23] is shown for the four promoter classes and all promoters. Click here for file

Additional file 7 **Relationship between transcriptional plasticity and Pol II occupancy around TSS**. All genes were divided into five groups according to their average transcriptional plasticity, and the average Pol II occupancy [33] around TSS (from -100 to +100) was shown for each group. Error bars were calculated by bootstrapping. Click here for file
