# Supplementary figures and images for: Insights into distinct regulatory modes of nucleosome positioning
Source: BMC Genomics. 2009 Dec 14;10:602. doi: 10.1186/1471-2164-10-602 (PMC2799443; doi:10.1186/1471-2164-10-602)

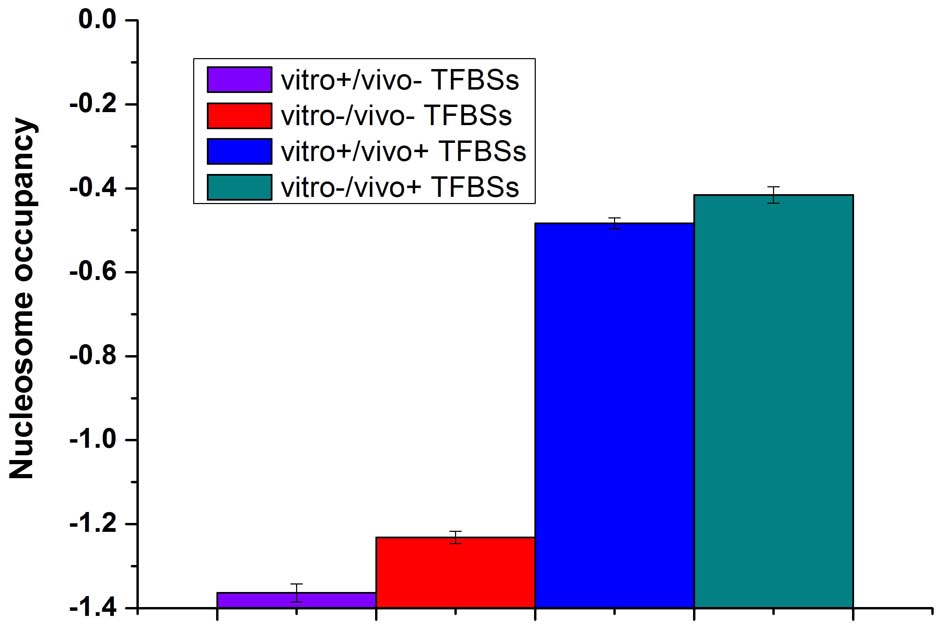

Supplement: Additional file 1 — In vivo nucleosome occupancy by Lee et al. for the four TFBS classes. Average values that quantify the levels of in vivo nucleosome occupancy [1] are shown for the four TFBS classes. We calculated for each TFBS the average nucleosome occupancy over the region it covers. Error bars were calculated by bootstrapping. [file 1471-2164-10-602-S1.JPEG]

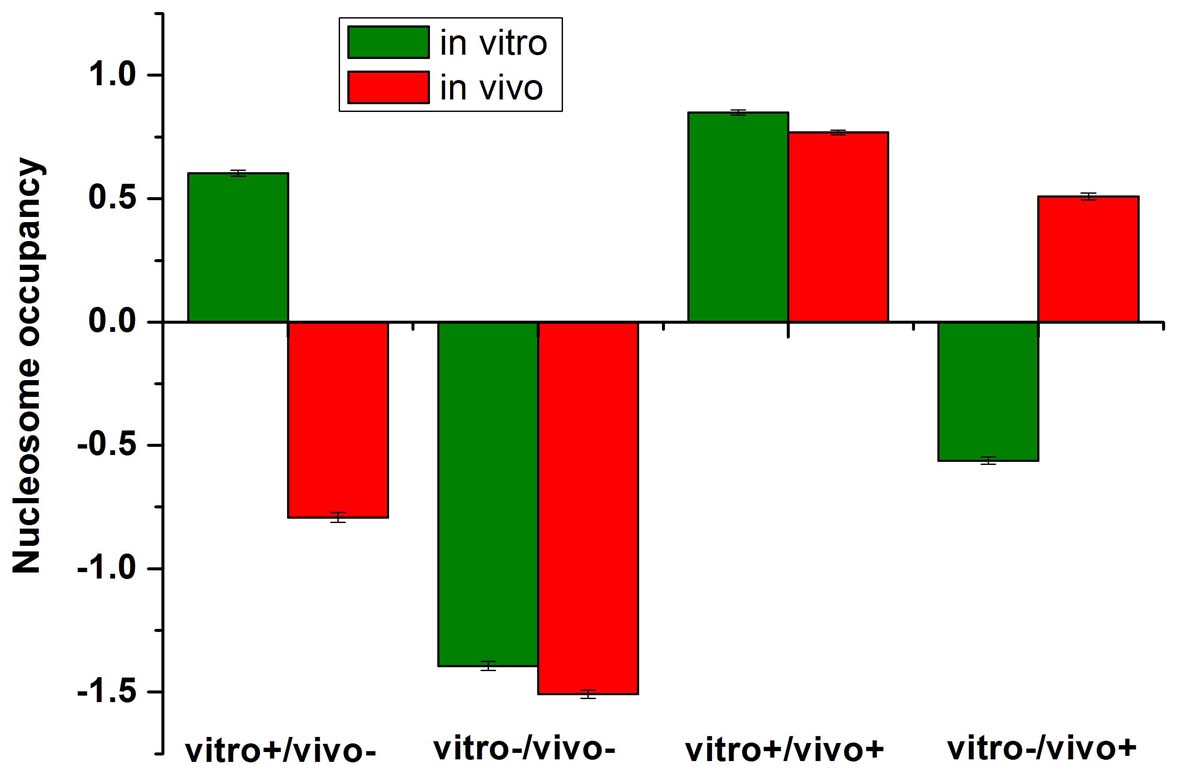

Supplement: Additional file 2 — In vitro and in vivo nucleosome occupancy by Kaplan et al. for the four TFBS classes. The average in vitro and in vivo nucleosome occupancy [23] over each TFBS was computed, respectively. The average resulting values are shown for the four TFBS classes (1,378 vitro+/vivo- TFBSs, 4,235 vitro-/vivo- TFBSs, 2,377 vitro+/vivo+ TFBSs, 980 vitro-/vivo+ TFBSs). Error bars were calculated by bootstrapping. [file 1471-2164-10-602-S2.JPEG]

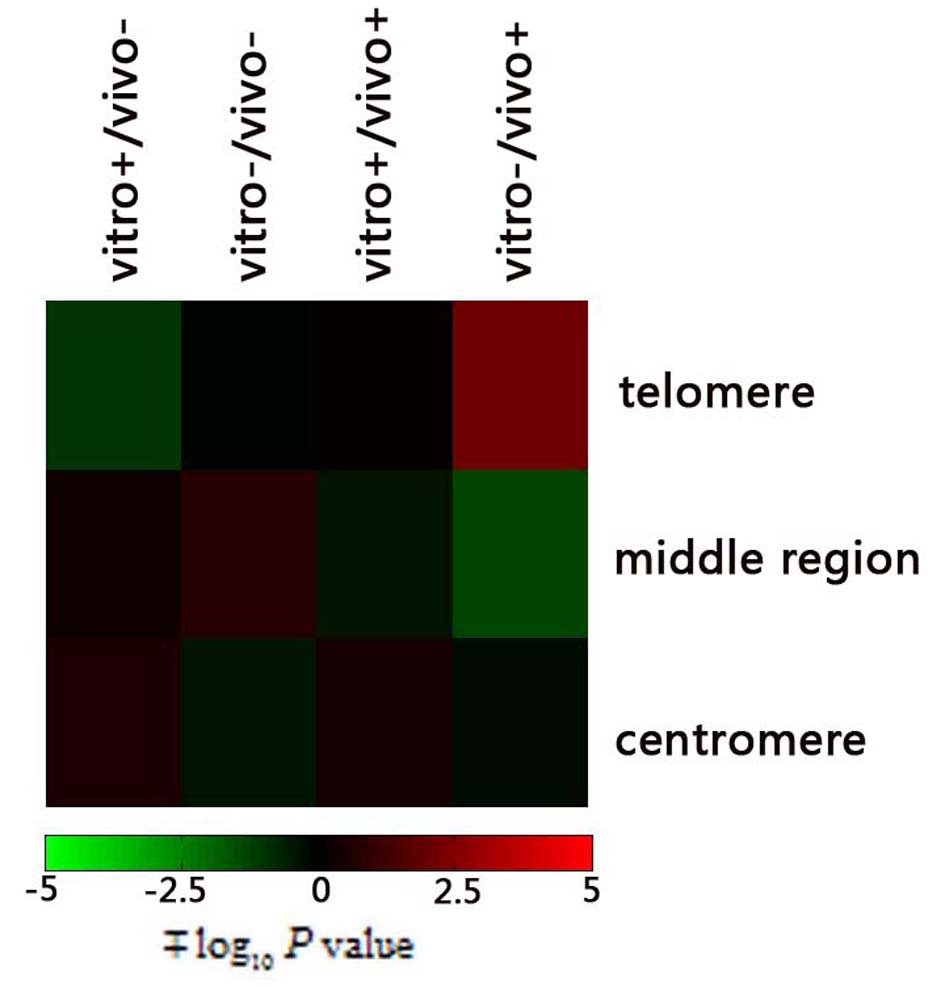

Supplement: Additional file 3 — Regional preference and dispreference for each TFBS class. Rows represent the 3 regions labelled telomere (the ends of chromosomes, 5% of the chromosomes), centromere (taken from SGD [37]) and the middle region between centromere and telomere. Each column represents the μ log10 P value (negative sign indicates that the number of TFBSs is higher than expected (i.e. preference), while positive sign indicates that the number of TFBSs is lower than expected (i.e. dispreference)) significance profile of a specific TFBS class. For each of the four TFBS classes, we first counted the numbers of TFBSs that are on each of the 3 regions and on the other 2 regions, respectively. We next counted the total numbers of all TFBSs that are on each of the 3 regions and on the other 2 regions, respectively. By using chi-test to evaluate the overlap in membership of specific class of TFBSs (observed occurrence) with the collection of all TFBS classes (expected occurrence) that reside on the specific region and on the other regions, we evaluated whether the particular TFBS class shows a strong preference or avoidance to reside on specific region. P values were calculated using the CHITEST formula in Excel. Using the threshold (P < 0.01) to assess the statistical significance, we found that only vitro-/vivo+ TFBSs showed a slight preference to telomere (P = 0.008). The other three TFBS classes did not show a preference or avoidance to reside on specific regions on the chromosomes. [file 1471-2164-10-602-S3.JPEG]

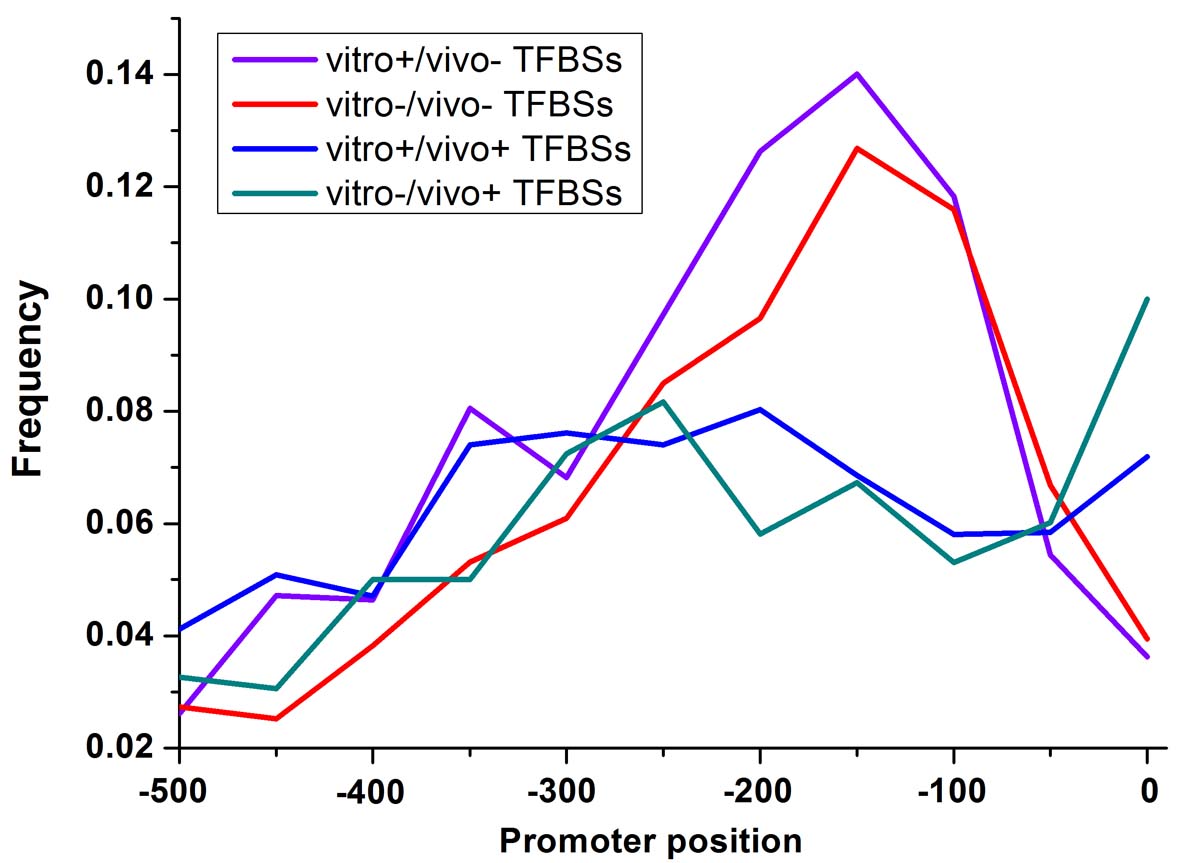

Supplement: Additional file 4 — Distribution of TFBSs at promoter regions. Distribution of TFBSs relative to the ATG start codon of an ORF is shown for the four TFBS classes. [file 1471-2164-10-602-S4.JPEG]

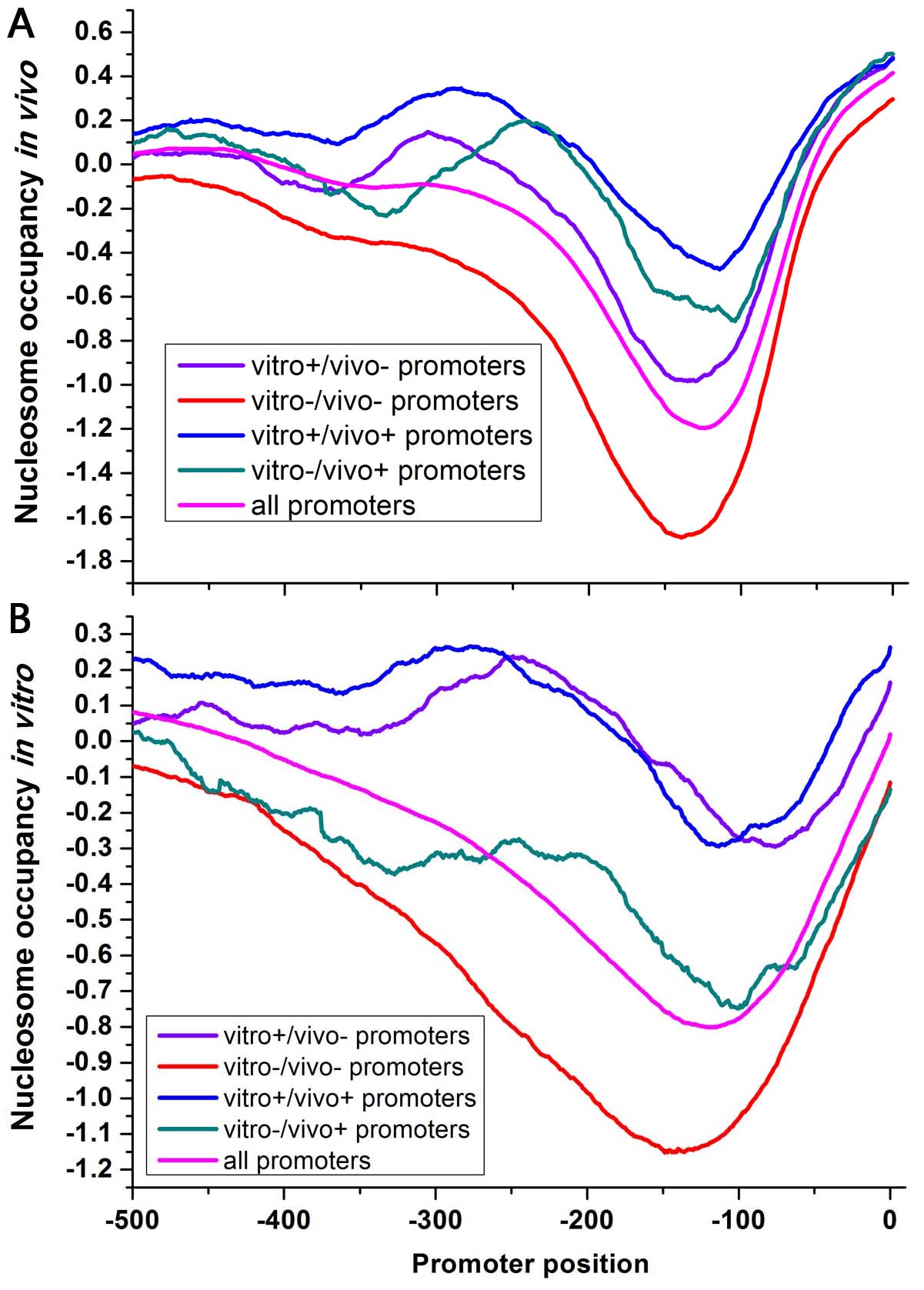

Supplement: Additional file 6 — Average nucleosome occupancy at promoter regions. (A) Average nucleosome occupancy in vivo [23] is shown for the four promoter classes and all promoters. (B) Average nucleosome occupancy in vitro [23] is shown for the four promoter classes and all promoters. [file 1471-2164-10-602-S6.JPEG]

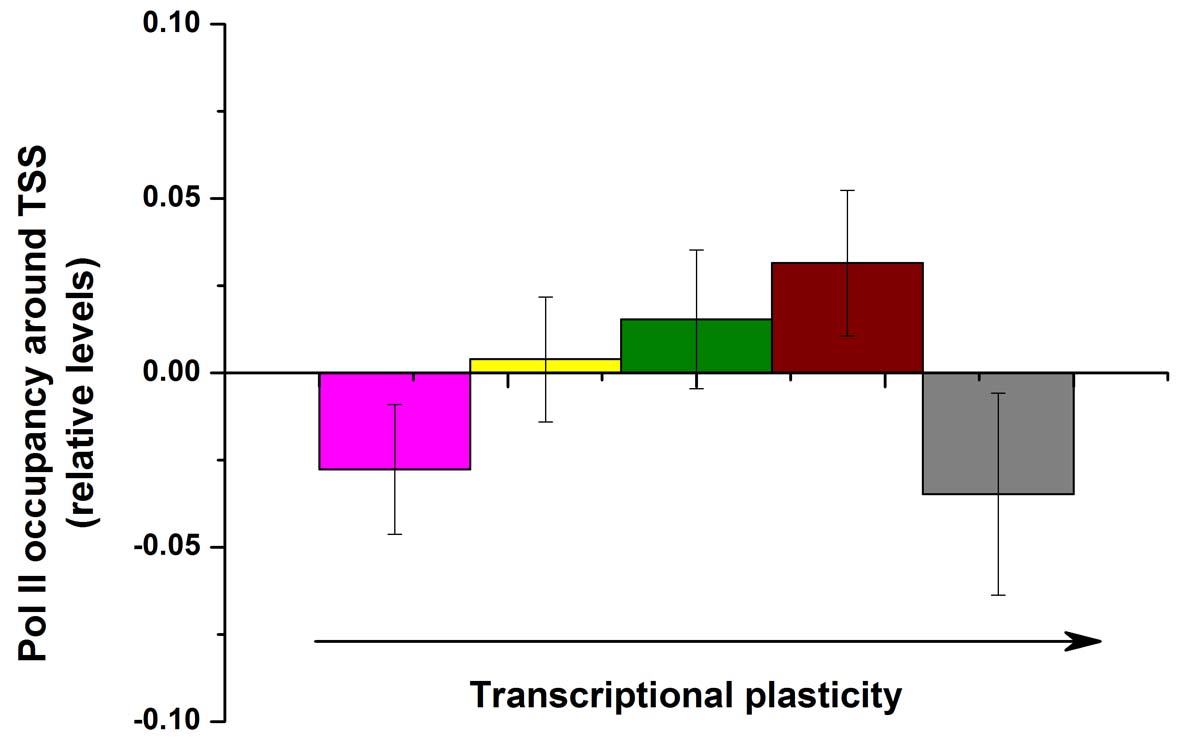

Supplement: Additional file 7 — Relationship between transcriptional plasticity and Pol II occupancy around TSS. All genes were divided into five groups according to their average transcriptional plasticity, and the average Pol II occupancy [33] around TSS (from -100 to +100) was shown for each group. Error bars were calculated by bootstrapping. [file 1471-2164-10-602-S7.JPEG]
